# Home Treatment for Catarrhs and Colds

**Published:** 1895-04

**Authors:** 


### Book Reviews.

---

HOME TREATMENT FOR CATARRHS AND COLDS. By Leonard A. Dessar, M. D., Visiting Laryngologist to St. Mark's Hospital, and to Mt. Sinai Hospital Dispensary, Etc., Etc. New York: Home Serus Publishing Co.

The above is a neat little volume that deals more with the laity than with professional readers. To those who wish the treatments, etc. of these diseases "boiled down," there is no handier guide than this little volume. It has forty-five handsome illustrations, and is printed with clear, large type on good paper. G. B.

